# An updated systematic review of epidemiological evidence on hormonal contraceptive methods and HIV acquisition in women

**DOI:** 10.1097/QAD.0000000000001228

**Published:** 2016-10-25

**Authors:** Chelsea B. Polis, Kathryn M. Curtis, Philip C. Hannaford, Sharon J. Phillips, Tsungai Chipato, James N. Kiarie, Daniel J. Westreich, Petrus S. Steyn

**Affiliations:** a Guttmacher Institute, New York, New York; b Department of Epidemiology, Johns Hopkins Bloomberg School of Public Health, Baltimore, Maryland; c Division of Reproductive Health, Centers for Disease Control and Prevention (CDC), Atlanta, Georgia, USA; d Centre of Primary Academic Care, University of Aberdeen, United Kingdom; e Department of Family Medicine, Boston University School of Medicine/Boston Medical Center, Boston, Massachusetts, USA; f Department of Obstetrics and Gynecology, University of Zimbabwe College of Health Sciences, Harare, Zimbabwe; g Department of Reproductive Health and Research, World Health Organization (WHO), Geneva, Switzerland; h Department of Epidemiology, University of North Carolina, Chapel Hill, North Carolina, USA.

**Keywords:** contraceptive implants, depot medroxyprogesterone acetate, HIV acquisition, hormonal contraception, injectable contraception, norethisterone enanthate, oral contraception, systematic review

## Abstract

**Objective and design:**

Some studies suggest that specific hormonal contraceptive methods [particularly depot medroxyprogesterone acetate (DMPA)] may increase women's HIV acquisition risk. We updated a systematic review to incorporate recent epidemiological data.

**Methods:**

We searched for articles published between 15 January 2014 and 15 January 2016 and hand-searched reference lists. We identified longitudinal studies comparing users of a specific hormonal contraceptive method against either nonusers of hormonal contraception or users of another specific hormonal contraceptive method. We added newly identified studies to those in the previous review, assessed study quality, created forest plots to display results, and conducted a meta-analysis for data on DMPA versus non-use of hormonal contraception.

**Results:**

We identified 10 new reports of which five were considered ‘unlikely to inform the primary question’. We focus on the other five reports, along with nine from the previous review, which were considered ‘informative but with important limitations’. The preponderance of data for oral contraceptive pills, injectable norethisterone enanthate, and levonorgestrel implants do not suggest an association with HIV acquisition, though data for implants are limited. The new, higher quality studies on DMPA (or nondisaggregated injectables), which had mixed results in terms of statistical significance, had hazard ratios between 1.2 and 1.7, consistent with our meta-analytic estimate for all higher quality studies of hazard ratio 1.4.

**Conclusion:**

Although confounding in these observational data cannot be excluded, new information increases concerns about DMPA and HIV acquisition risk in women. If the association is causal, the magnitude of effect is likely hazard ratio 1.5 or less. Data for other hormonal contraceptive methods, including norethisterone enanthate, are largely reassuring.

## Introduction

Empowering women and couples with the tools necessary to prevent unintended pregnancy and avoid sexually transmitted infections including HIV is critically important for individual and public health. Hormonal contraceptive methods are highly effective for prevention of unintended pregnancy and associated sequelae. However, some epidemiological studies suggest an association between use of specific hormonal contraceptive methods [particularly depot medroxyprogesterone acetate (DMPA)] and an increased risk of HIV acquisition in women; other studies have not reported this association [1]. This question is critically important for women's health, particularly in sub-Saharan Africa, where high rates of HIV coincide with high use of injectable contraception [2]. Many regions with high HIV prevalence also have high rates of unmet need for contraception, unintended pregnancy, and maternal mortality and morbidity, underlying the imperative for access to effective contraception [3,4].

Several biologically plausible mechanisms have been postulated to explain how various hormonal contraceptive methods could increase women's risk of HIV acquisition, including possible disruption of epithelial barriers, alterations in immune cell populations, or soluble inflammatory responses [5–8]. The effect of hormonal contraception on cervical immunity is influenced by the genital tract microenvironment and presence of infections [9]. Interpretation of current data on biologic and immunologic impacts from hormonal contraceptive use is hampered by studies that fail to account for different hormones, diverse dosages, and hormonal contraceptive delivery routes [7]. Women using particular hormonal contraceptive methods may also have other characteristics (e.g. different patterns of condom use), which could impact HIV acquisition risk.

A previous systematic review of epidemiological evidence assessed all relevant evidence published prior to 15 January 2014 [1]. The review was conducted independently of the WHO guidance development process and served as an input into WHO deliberations related to updating the medical eligibility criteria for contraceptive use (refer to Appendix A, for current WHO guidance for hormonal contraceptive use among women at high risk of HIV) [10]. Given the public health importance of this topic, we updated our previous systematic review to incorporate newly published, pertinent epidemiological evidence.

## Methods

We conducted this systematic review according to Preferred Reporting Items for Systematic Reviews and Meta-Analyses guidelines [11].

### Inclusion/exclusion criteria

We included published primary research reports on women who were HIV-negative at baseline in longitudinal studies (observational studies or randomized trials, or meta-analyses containing data not otherwise captured in our search strategy) that measured incident, laboratory-confirmed HIV infection among women who used a specific method of hormonal contraception [injectables, oral contraceptives, implants, patches, rings, or levonorgestrel intrauterine devices (LNG-IUDs)] compared with incident HIV infections among women using a nonhormonal contraceptive method (e.g. condoms, nonhormonal IUD, sterilization, withdrawal, etc.) or no contraceptive method (henceforth, ‘hormonal contraceptive versus non-use of hormonal contraception’ comparisons). Some studies compared hormonal contraceptive users against a heterogeneous group including other hormonal contraceptive users, nonhormonal method users, and nonusers of contraception. We identified and included such studies, but considered the composition of the comparison group when assessing study quality.

We also included studies comparing incident HIV infection among HIV-negative women using a specific method of hormonal contraception against HIV-negative women using another specific method of hormonal contraception (henceforth, ‘head-to-head’ analyses) in which the comparison group did not contain nonhormonal method users or nonusers of contraception.

We excluded studies that did not report a risk estimate for the relationship between hormonal contraceptive use and HIV acquisition, cross-sectional studies, studies assessing only emergency contraception, conference abstracts, or other unpublished reports.

### Search strategy

We retained all articles included in the previous systematic review, unless superseded by a new published analysis based upon the same data. We searched PubMed and Embase (Appendix B) for articles published in any language between 15 January 2014 and 15 January 2016, inclusive. We hand-searched reference lists of included studies. C.B.P. conducted the literature search and C.B.P., K.M.C., and P.C.H. screened titles, abstracts, and full-text manuscripts to determine inclusion using Covidence software [12].

### Data extraction and quality assessment

We applied a study quality assessment framework used in our 2014 systematic review, with slight modifications for clarity [1]. Briefly, studies that did not include adjustment for condom use or which had unclear measurement of exposure to hormonal contraception (refer to Appendix C, for a full explanation of the quality assessment criteria) were considered ‘unlikely to inform the primary question’. For comprehensiveness, we included all studies that met our inclusion criteria, regardless of quality. However, we focused on studies with neither of the two quality concerns noted above; we considered these studies ‘informative but with important limitations’ (IBWILs) to acknowledge that all studies to date are vulnerable to residual or uncontrolled confounding. All authors participated in confirming the study quality assessment framework and in rating the quality of each study. We adapted previously used abstraction forms that were pilot tested by all coauthors. All coauthors abstracted data from each newly included study that was considered as IBWIL. We contacted study investigators if clarifications were needed.

### Graphical summaries

We created forest plots using Microsoft Excel 2013 (Microsoft, Redmond, Washington, USA) to summarize point estimates for a given contraceptive method [i.e. oral contraceptives, injectables (nonspecified, DMPA, and norethisterone enanthate (NET-EN)), or implants]. We focus on graphics summarizing only studies considered IBWIL, but graphs depicting all studies regardless of quality are provided in Appendix D.

Most studies estimated hazards ratios using Cox proportional hazards models; some also included estimates from a marginal structural model (MSM) (for additional discussion, refer to [1,13]). A few estimated only incidence rate ratios (IRRs) (Tables 1 and 2). For clarity of presentation, we display the IRR or Cox hazards ratio, unless the MSM model generated qualitatively different estimates, in which case both Cox and MSM estimates are shown.

As in 2014, we requested disaggregated estimates from authors of new studies classified as IBWIL and which included women from South Africa (where use of both DMPA and NET-EN is common) but which did *not* report separate estimates for each. Disaggregated estimates have reduced statistical power but greater epidemiological and clinical value, given the potential for different biological effects by contraceptive type or formulation.

### Meta-analysis

Given concerns specific to DMPA, we performed a statistical meta-analysis for the effect of DMPA versus non-use of hormonal contraception on HIV acquisition (studies that did not disaggregate injectables were not included). For maximum comparability, we included the most fully adjusted Cox hazards ratio estimates from each study, except one that reported an adjusted IRR (IRRs can be interpreted similarly to hazards ratios under certain conditions [14]). We log-transformed reported adjusted point estimates and 95% confidence intervals (95% CIs) to calculate standard errors using a random effects model [15]. We assessed statistical heterogeneity using the *I*^2^ statistic [16]. Analyses were performed using Stata (Version 13.1, College Station, Texas, USA).

## Results

### Description of included studies

**Fig. 1:**
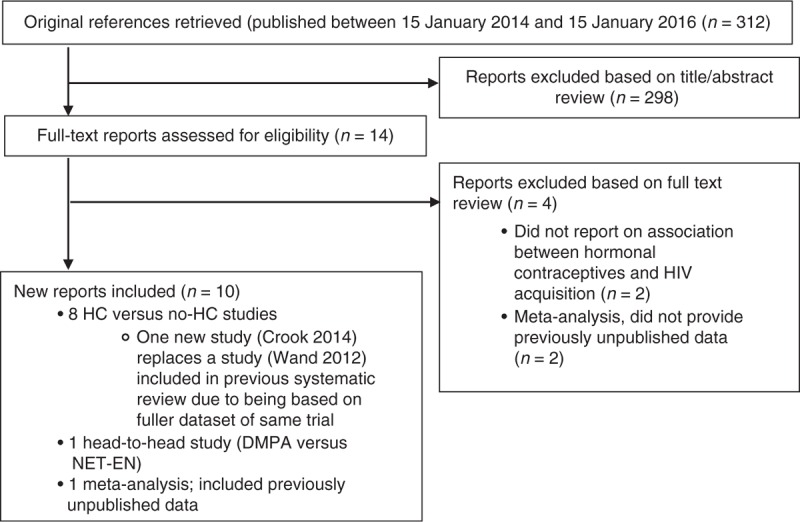
Identification of newly included studies.

Twenty-two studies were included in our previous review [1]. For this review, we screened 312 new references, assessed 14 full-text reports, and excluded four: two did not report on the association of interest [17,18] and two meta-analyses contained published data already captured by our search strategy (including them would have resulted in double-counting of data, instead they are mentioned in our discussion) (Fig. 1) [19,20].

We relied upon the search from a previous systematic review [1] to identify all relevant studies published prior to 15 January 2014. We conducted our title/abstract review using Covidence online software [12].

We included 10 new reports [21–30]; one [21] superseded a previously included study [31]. A large, individual participant data (IPD) meta-analysis [26] used raw data from 18 datasets, including seven not previously utilized to investigate the association of interest [17,32–37]. To incorporate the previously unpublished information (while avoiding double-counting from previously published studies), we requested a subanalysis restricted to data from these seven studies in a hormonal contraceptive versus non-use of hormonal contraception comparison [38]. The IPD meta-analysis also included a head-to-head comparison that none of our included component studies had assessed; here we used results from the original article [26].

Table 1 describes 10 newly included studies; information on previously included studies is available elsewhere [1]. A total of 31 studies (comprising 34 reports) were included [21–30,39–62]. Thirty assessed hormonal contraceptive versus non-use of hormonal contraception comparisons [21–29,39–62] and two assessed head-to-head comparisons [26,30].

Among 30 studies with hormonal contraceptive versus non-use of hormonal contraception comparisons, 24 included estimates specific to (or largely composed of) oral contraceptives [21–24,26,27,29,39,41–46,49–53,55–59,61,62]. Twenty-four included estimates specific to (or largely composed of) injectables [21,24–29,39–48,50–53,55,56,58,60,62] and three included implant-specific estimates [27,39,50,54]. All studies assessing DMPA assessed intramuscular DMPA, rather than the lower dose, subcutaneous formulation. No study assessed contraceptive patches, rings, combined injectables, or LNG-IUDs. Among two head-to-head studies, two compared DMPA versus NET-EN [26,30] and one compared DMPA versus combined oral contraceptives (COCs) and NET-EN versus COCs [26].

### Hormonal contraceptive versus non-use of hormonal contraception studies considered informative but with important limitations

Of 30 hormonal contraceptive versus non-use of hormonal contraception, we rated 12 as IBWIL [21,26,27,29,39,42,43,47,51–53,55,56,58], including four newly identified studies [21,26,27,29]. Table 2 provides details on new IBWIL studies; information on previously included IBWIL studies is available elsewhere [1]. The four new studies included a large IPD meta-analysis that assessed oral contraceptives, DMPA, and NET-EN across a range of datasets [26], an analysis from an 18-year cohort study of Zambian serodiscordant couples to assess oral contraceptives, DMPA, and implants [27], and two analyses from large microbicide trials, one assessing unspecified injectables [29] and the other assessing oral contraceptives, DMPA, and NET-EN [21]. Below, we summarize results from all 12 hormonal contraceptive versus non-use of hormonal contraception studies considered IBWIL. Readers should consult the relevant tables and figures for additional detail (such as 95% CIs); descriptions below provide a succinct synthesis of the overall evidence base. We discuss studies according to whether results were significant at *P* less than 0.05, but acknowledge that, considered alone, *P* values are an imperfect indicator of significance [63].

#### Implants

Neither of two IBWIL studies assessing levonorgestrel-based implants (Norplant or Jadelle) [27,39,54] suggested a statistically significant increased risk of HIV. Point estimates ranged from adjusted hazards ratio (adjHR) 0.96 to 1.60; 95% CIs were wide.

#### Oral contraceptives

**Fig. 2:**
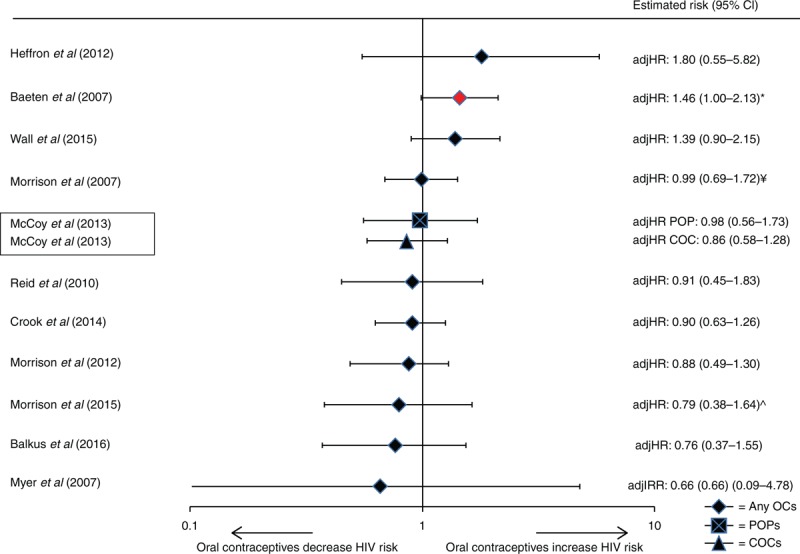
Use of oral contraceptives (versus non-use of hormonal contraception) and HIV acquisition, among 11 studies considered informative but with important limitations.

Of 11 IBWIL studies assessing oral contraceptives [21,26,27,29,38,39,42,51–53,55,56,58], one reported a marginally significant increase in risk (adjHR: 1.46, *P* = 0.05); 10 reported nonsignificant estimates ranging from adjusted incidence rate ratios (adjIRR) 0.66 to adjHR 1.80 (Fig. 2). One study disaggregated COCs and progestin-only pills (POPs); point estimates were similar and nonsignificant (adjHR: 0.86 and 0.98, respectively) [51].

Error bars show 95% confidence intervals. Studies are arranged in order of decreasing magnitude of risk estimate, except if a single study disaggregated progestin-only pills and combined oral contraceptives, in which the case both estimates are adjacent (as indicated by a box around the study identifiers). Graph does not display estimates from marginal structural models. adjIRR, adjusted incidence risk ratio; adjHR, adjusted hazard ratio. ^∗^Analysis showed significant findings at *P* = 0.05 (marker also displayed in red). ^^^Unpublished estimates from a subanalysis of Morrison *et al.*[26] meta-analysis, restricted to pooled analysis using databases not previously used to publish estimates on hormonal contraceptive methods and HIV acquisition risk.

#### Injectables

**Fig. 3:**
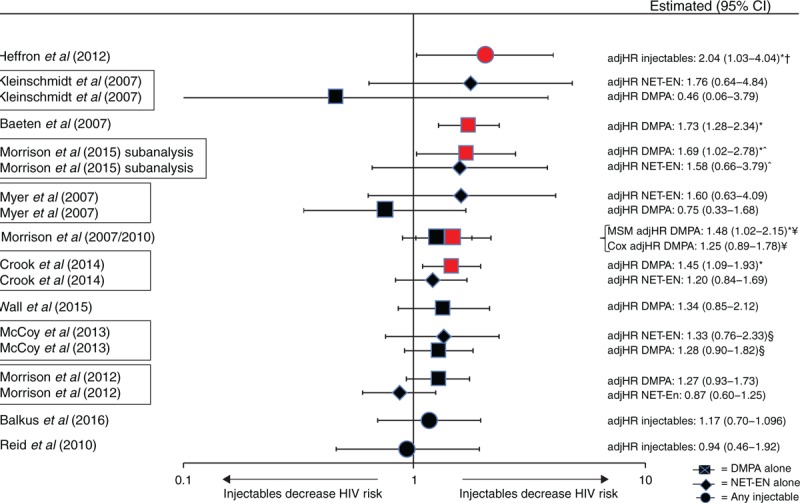
Use of injectables (depot medroxyprogesterone acetate, norethisterone enanthate, or unspecified injectable) versus non-use of hormonal contraception and HIV acquisition, among 12 studies considered informative but with important limitations.

**Fig. 4:**
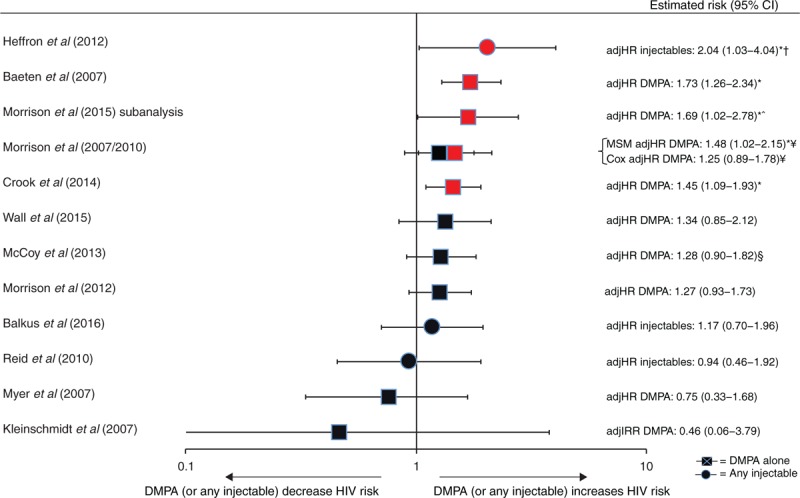
Use of depot medroxyprogesterone acetate (or unspecified injectable) versus non-use of hormonal contraception and HIV acquisition, among 12 studies considered informative but with important limitations.

**Fig. 5:**
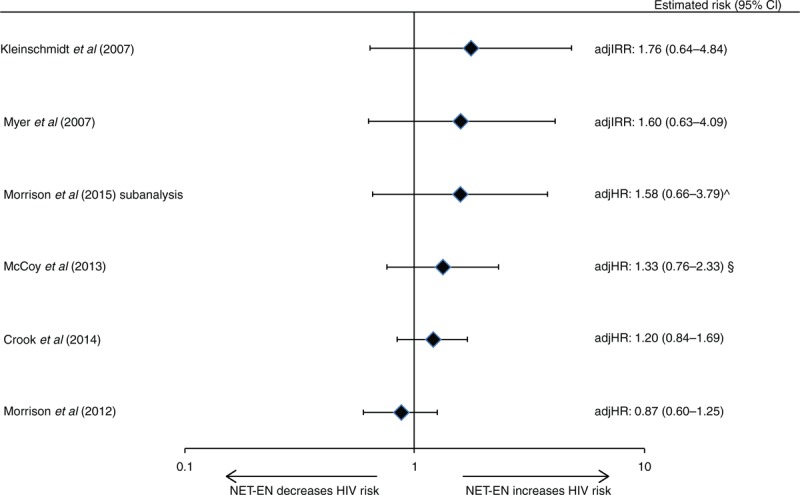
Use of norethisterone enanthate versus non-use of hormonal contraception and HIV acquisition, among six studies considered informative but with important limitations.

Of 12 IBWIL studies assessing injectables (DMPA, NET-EN, or a mix of both) [21,26,27,29,38,39,42,47,51–53,55,56,58], nine provided DMPA-specific estimates and three provided estimates for unspecified injectables. Five studies reported a statistically significant increase in risk with either unspecified injectables [42] or DMPA [21,26,38,39,52,53], although the point estimate in one was not statistically significant in a Cox proportional hazards model [53] (Figs. 3 and 4). Point estimates from Cox models from these five studies ranged from adjHR 1.45 to 2.04 (Figs. 3 and 4) [21,26,38,39,42,52,53]; the largest estimate under an MSM model was 2.19 [42]. Among seven studies reporting nonstatistically significant results, point estimates ranged from adjIRR 0.46 to adjHR 1.34 (both DMPA-specific) [27,29,47,51,55,56,58]. None of six studies assessing NET-EN reported statistically significant increases in HIV risk: point estimates ranged from adjHR 0.87 to adjIRR 1.76 (Fig. 5) [21,26,38,47,51,55,56].

Error bars show 95% confidence intervals. Studies arranged in order of decreasing magnitude of risk estimate, except if a single study disaggregated depot medroxyprogesterone acetate and norethisterone enanthate, in which the case both estimates are adjacent (as indicated by a box around the study identifiers). Graph does not display estimates from marginal structural models, except where use of such models resulted in different conclusion regarding statistical significance; in such cases, estimates from both models are displayed on a single line (also identified by bracket signs). *Note:* displays all data on injectables (depot medroxyprogesterone acetate, norethisterone enanthate, or unspecified). adjIRR, adjusted incidence risk ratio; adjHR, adjusted hazard ratio. ^∗^Analysis showed significant findings at *P* = 0.05 (marker also displayed in red). ^†^Estimate for Cox model taken from slightly updated analysis which controlled for total number of unprotected sex acts. ^^^Unpublished estimates from a subanalysis of Morrison *et al.*[26] meta-analysis, restricted to pooled analysis using databases not previously used to publish estimates on hormonal contraceptive methods and HIV acquisition risk. ^¥^Different statistical models adjusted for slightly different confounders. ^§^Unpublished estimates disaggregated by injectable type.

Error bars show 95% confidence intervals. Studies arranged in order of decreasing magnitude of risk estimate. Graph does not display estimates from marginal structural models, except where use of such models resulted in different conclusion regarding statistical significance; in such cases, estimates from both models are displayed on a single line (also identified by bracket signs). adjIRR, adjusted incidence risk ratio. *Note:* restricts to all data on depot medroxyprogesterone acetate or unspecified injectables, as estimates of unspecified injectables are likely comprised largely of DMPA. adjHR, adjusted hazard ratio. ^∗^Analysis showed significant findings at *P* = 0.05 (marker also displayed in red). ^†^Estimate for Cox model taken from slightly updated analysis which controlled for total number of unprotected sex acts. ^^^Unpublished estimates from a subanalysis of Morrison *et al.*[26] meta-analysis, restricted to pooled analysis using databases not previously used to publish estimates on hormonal contraceptive methods and HIV acquisition risk. ^¥^Different statistical models adjusted for slightly different confounders. ^§^Unpublished estimates disaggregated by injectable type.

Error bars show 95% confidence intervals. Studies arranged in order of decreasing magnitude of risk estimate. Graph does not display estimates from marginal structural models. adjIRR, adjusted incidence risk ratio; adjHR, adjusted hazard ratio. ^^^Unpublished estimates from a subanalysis of Morrison *et al.*[26] meta-analysis, restricted to pooled analysis using databases not previously used to publish estimates on hormonal contraceptive methods and HIV acquisition risk. §Unpublished estimates disaggregated by injectable type.

### Head-to-head studies considered informative but with important limitations

**Fig. 6:**
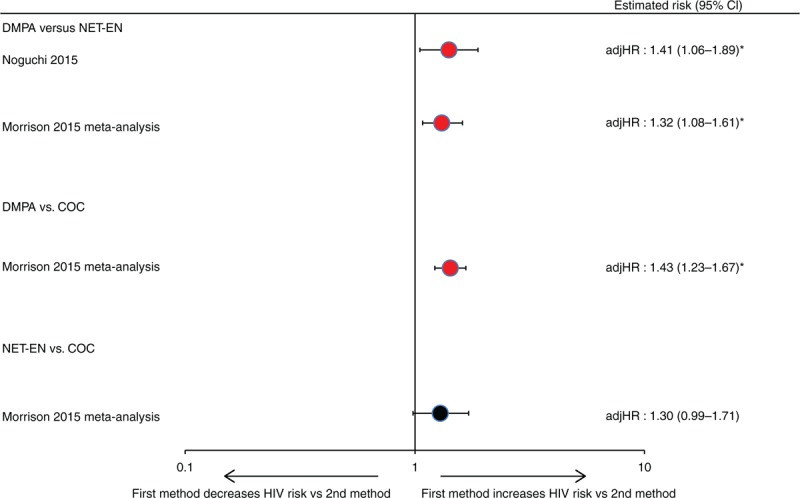
Hormonal contraceptive methods and HIV acquisition in head-to-head studies, among two studies considered informative but with important limitations.

No head-to-head comparison studies were available in the previous review [1]. Both newly included head-to-head studies were considered IBWIL (Tables 1 and 2) [26,30]. Both reported a statistically significant increased risk of HIV for DMPA use (adjHR: 1.32 and 1.41) versus NET-EN use [26,30]. The IPD meta-analysis also compared each injectable against COCs, reporting significantly increased risk for DMPA versus COCs (adjHR: 1.43, 95% CI: 1.23–1.67) and a borderline nonsignificant increased risk for NET-EN versus COCs: adjHR 1.30 (0.99–1.71) (Fig. 6) [26].

Error bars show 95% confidence intervals. Within each comparison group, studies are arranged in order of decreasing magnitude of risk estimate. adjHR, adjusted hazard ratio. ^∗^Analysis showed significant findings at *P* = 0.05 (marker also displayed in red).

### Meta-analysis

Ten estimates, from nine published studies with DMPA-specific estimates versus non-use of hormonal contraception [21,27,39,42,47,51,53,55,56] and a subanalysis of previously unpublished information from an IPD meta-analysis [26], were included in our meta-analysis of the effect of DMPA on HIV acquisition (Appendix D, Fig. 5,). The overall effect estimate was 1.40 (95% CI: 1.23–1.59) with an *I*^2^ of 0%, indicating minimal quantitative heterogeneity.

### Effect modification

One study reported increased HIV risk with DMPA and oral contraceptives in younger (18–24 years) but not older women [52]; eight studies reported no effect modification by age [21,27,29,39,42,47,51,56]; most studies reported no effect modification by herpes simplex virus type 2 (HSV-2) status [21,29,39,42,51], whereas two reported effect modification in opposite directions [One observed higher HIV risk with DMPA in HSV-2 seronegative women (Morrison *et al.*[52]) and the other observed higher HIV risk with DMPA (versus NET-EN) in HSV-2 seropositive women (Noguchi *et al.*[30]).]. Two studies reported no effect of modification by study site [21,30], one reported greater risk for oral contraceptives and DMPA in a Ugandan site versus a Zimbabwean site [53]. A study in serodiscordant couples reported no effect modification for genital ulceration, inflammation, viral load of HIV-positive partner at baseline, or fertility intentions [27]. Another study reported no effect modification by reported condom use at baseline, participant behavioral risk, or prevalent chlamydia or gonorrhea [53].

Within the IPD meta-analysis, assessment for effect modification was conducted with information from all 18 studies (some of which were also included in our review). No evidence of interaction was reported with any method for age (15–24 versus >25 years), HSV-2 status at baseline, or HIV incidence in population (low versus high) [26]. Increased HIV risk was observed for COC use in East Africa (adjHR: 1.58, 95% CI: 1.19–2.09) but not South Africa or Southern Africa, and for DMPA use in east and South Africa (adjHR: 2.09, 95% CI: 1.68–2.80; adjHR: 1.30, 95% CI: 1.11–1.53), but not Southern Africa. Populations that reported engaging in transactional sex work had an increased HIV risk with COCs (adjHR: 1.51, 95% CI: 1.09–2.10) unlike populations without transactional sex work. Finally, smaller point estimates were observed among studies deemed by the investigators as at lower risk of methodological bias: adjHR for DMPA: 1.22 (95% CI: 0.99–1.50) and adjHR for NET-EN: 0.67 (95% CI: 0.47–0.96). Table 3 details how the IPD meta-analysis investigators defined lower risk of bias in comparison with our quality criteria.

## Discussion

### Interpretation of overall results

As in our 2014 review, current data do not suggest an increased risk of HIV acquisition among women using oral contraceptives [1]. Extremely limited data do not suggest a statistically significant increased risk of HIV acquisition among users of levonorgestrel implants; no data are available regarding etonogestrel implants. In 2014, one of five studies that was considered IBWIL suggested an increased risk of HIV acquisition with NET-EN injectables [31]. In this updated review, that study was replaced by a larger, more sophisticated analysis of the same dataset [21], and increased HIV risk was no longer observed. Thus, currently available data for injectable NET-EN use do not suggest an association with HIV acquisition in women.

Although concerns around confounding in observational data remain relevant, newly available evidence regarding injectable DMPA use increases concern about a potential causal association with HIV acquisition. Twelve studies considered IBWIL assessed DMPA or nondisaggregated injectables compared with non-use of hormonal contraception; four or five (depending on the statistical model considered) reported statistically significant increased risks of HIV acquisition, ranging from adjHR 1.45 to 2.04 in Cox models (or 2.19 in MSM models). Among four newly included studies, two reported statistically significant increased risk (adjHR: 1.45 and 1.69), including one very large study [21] and a subanalysis of a large IPD meta-analysis [26]. A smaller study among serodiscordant couples [27] reported a nonsignificant estimate of adjHR 1.34, and data from a microbicide trial also had a nonsignificant estimate of adjHR 1.17 but did not disaggregate between injectables [29]. Head-to-head comparisons were newly available and may be less confounded by unmeasured or residual behavioral differences than comparisons from hormonal contraceptive versus non-use of hormonal contraception studies, particularly if groups compared in head-to-head studies use different types of the same delivery method (i.e., injectable DMPA versus injectable NET-EN) [30]. A head-to-head analysis of VOICE data reported a 41% increased risk of HIV acquisition in DMPA versus NET-EN users [30]. In the IPD meta-analysis [26], DMPA use was associated with a significantly increased risk of HIV acquisition of 30–40% when compared with either NET-EN or COC use. Comparing NET-EN against COC users suggested higher risk with NET-EN, though this was not statistically significant at *P* less than 0.05 (*P* = 0.055). Although residual confounding cannot be ruled out in any observational study, several recently published studies suggesting an increased risk of HIV acquisition among DMPA users had few limitations apart from being observational (Table 2).

Recent analyses contradict the hypothesis that differential over-reporting of condom use by hormonal contraceptive users explains observed associations between hormonal contraceptive use and HIV infection in some studies [64]. However, the possibility remains that certain confounders are specific to DMPA users. In South Africa (where both DMPA and NET-EN injectables are used), studies suggest that women perceive DMPA and NET-EN differently, and providers may preferentially prescribe different injectable types to specific subpopulations, which could result in confounding specific to injectable type [30,65,66]. Although beyond the scope of this review, emerging evidence related to DMPA and HSV-2 acquisition must also be considered [67,68].

Taken together, the new evidence points toward heightened concerns that the association between DMPA use and HIV acquisition may not be fully explained by confounding or other methodological problems. In contrast, additional reassuring evidence of no significant association for other hormonal contraceptive methods (oral contraceptives, NET-EN, and implants) is newly available. If the association between DMPA and HIV acquisition risk is causal, meta-analyses, including our own, suggest a likely increase in risk of hazards ratio 1.5 or less.

The quality of epidemiological evidence on this issue has improved over time. Several newly published studies used recent analytic recommendations [13] or other innovative analytic techniques. For example, Crook *et al.*[21] conducted a particularly thorough exploration of statistical methodology and incorporated multiple sensitivity analyses to assess the robustness of their findings, Morrison *et al.*[26] contributed substantial new data in a carefully conducted IPD meta-analysis, and Noguchi *et al.*[30] examined an alternative comparison group (NET-EN users).

The methodological contribution of three newly published meta-analyses varied. In addition to the IPD meta-analysis included in our review [26], two meta-analyses [19,20] utilized data already included as primary studies in our systematic reviews (thus, adding no information beyond that already included in this review). Although all three meta-analyses reported summary estimates for DMPA similar to our own (hazards ratio 1.4–1.5), one of the excluded meta-analyses contained no assessment of study quality and included several studies with serious methodological limitations [20], which raises particular concern in the context of meta-analysis of observational data (Table 3) [69]. Both excluded meta-analyses [19,20] double-counted [70] data by inclusion of both Wand and Ramjee [31] and Crook *et al.*[21]. We generated a meta-analytic estimate for DMPA, but recommend that such results be interpreted with caution, given the potential for spurious precision in meta-analyses of observational data [71]. The *I*^2^ value for our meta-analysis suggested minimal statistical heterogeneity, but qualitative differences between study populations and methods remain an important consideration [16]. That said, estimates from all four meta-analyses are similar, despite inclusion of slightly different component studies [26].

### Limitations

Previous reviews have addressed key methodological considerations about this body of literature, including potential for confounding, frequency, and accuracy of variable measurement, considerations related to ‘direct’ and ‘total’ effects, potential for publication bias, and limitations of individual studies, such as failure of some studies to disaggregate by specific hormonal content or formulation (e.g., most studies assessing oral contraceptives failed to disaggregate estimates by COCs or POPs) [1,72]. Our study quality framework is necessarily subjective, and we encourage continued discussion on how best to evaluate study quality in this body of evidence.

### Conclusion

There remain no data on use of contraceptive patches, rings, or hormonal IUDs and HIV acquisition in women. For implants, very limited data pertaining to levonorgestrel implants do not suggest increased risk, but more information is needed. In comparison, a larger amount of data are available for oral contraceptives and are generally reassuring. A growing number of studies have assessed injectable NET-EN, and although still limited, data are generally reassuring. For injectable DMPA, although some new, high-quality studies do not report a statistically significant increased risk of HIV acquisition, other new data, including studies directly comparing DMPA and NET-EN, tend to strengthen concerns about DMPA. If the association between DMPA and HIV acquisition risk is causal, data suggest a likely increase in risk of hazards ratio 1.5 or less. Several new studies have used recently proposed recommendations for analysis or other innovative methodological approaches [13], although as with all observational data, the possibility of uncontrolled or residual confounding remains. The growing, generally reassuring evidence about other hormonal contraceptive methods, including other injectables like NET-EN, stands in contrast to the DMPA-specific findings. An important next step is for WHO to determine whether these concerns warrant a reconsideration of global guidance for DMPA. Modeling studies can be useful in understanding net health impacts of various policy responses in different epidemiological contexts, including the risk of HIV, maternal mortality and morbidity, and access to alternative contraception and HIV prevention methods [2,73–76].

## Acknowledgements

We are grateful to Sharon Achilles for her thoughtful input related to describing potential biological mechanisms, and to all study investigators who provided additional information about their analyses. WHO provided support for the writing of this systmatic review and for the writing group to attend a working meeting in Geneva, Switzerland in October 2015. D.J.W. was partially funded by NIH DP2-HD-08-4070. The review was conducted independently of the WHO guidance development process; and conclusions represent the independent opinions of the authors. The findings and conclusions in this article do not necessarily reflect the positions and policies of the donor.

Role of authors: The World Health Organization (J.N.K. and P.S.S.) initiated the idea to conduct this systematic review update. C.B.P. led the conduct of the systematic review, including conducting the systematic literature search and drafting the manuscript. C.B.P., K.M.C., and P.C.H. screened titles, abstracts, and full-text manuscripts to determine study inclusion. S.J.P. conducted the statistical meta-analysis. All coauthors (C.B.P., K.M.C., P.C.H., S.J.P., T.C., J.N.K., D.J.W., and P.S.S.) participated in framing the study question, developing the quality criteria, abstracting study information and assessing study quality, interpreting the data, and contributing to the writing and editing of the manuscript.

Disclaimer: The findings and conclusions in this report are those of the authors and do not necessarily represent the official positions of the Guttmacher Institute, the Centers for Disease Control and Prevention, the World Health Organization, the National Institutes of Health, or other institutions with which the authors are affiliated.

### Conflicts of interest

C.B.P. worked at USAID between 2011 and 2014, led previous systematic reviews on this topic, and participated in preliminary discussions considering the ECHO trial. P.C.H. served as a reviewer for potential funders of the ECHO protocol to inform their funding decision. S.J.P. participated in the inception of the ECHO trial. T.C., J.N.K., and P.S.S. are current members of the ECHO trial consortium. We do not feel that participation in these activities influenced our work on this systematic review. K.M.C. and D.J.W. have nothing to declare.

## Supplementary Material

Supplemental Digital Content

## Figures and Tables

**Table 1:** Description of all newly included studies (published since 15 January 2014), ordered by publication year, for systematic review update on use of various hormonal contraceptive methods among women at risk of HIV acquisition.

First author, publication year, location	Design, purpose, period of data collection	Number enrolled, description of population	Results (point estimate [adjusted, where available] and 95% CIs)	Met criteria for being considered ‘informative but with important limitations’?
Direct evidence from HC versus no-HC studies				
Kapiga 2013 [24], Tanzania	Cohort; to assess feasibility, retention, and appropriateness of population for future HIV prevention trials. Recruitment 2008–2010	2229 women working in hotels, restaurants, bars, guesthouses or shops selling traditionally-brewed beer, or food-sellers at makeshift facilities in northern Tanzania	OCs and other non-DMPA HC (+/− condoms) adjIRR: 0.68 (0.23–2.04); DMPA (+/− condoms) adjIRR: 1.63 (0.75–3.52)	No, did not control for condom use (and selection process for inclusion of covariates in the statistical model was not based on assessing HC-HIV acquisition)
Dube 2014 [22], Mozambique (Beira)	Cohort; to measure HIV incidence in women at higher risk of HIV and assess the feasibility of recruiting and retaining them as research participants. Data collection 2009–2012	411 women enrolled (387 contributed follow-up data). HIV-seronegative women aged 18–35 with at least two sexual partners in the past month, recruited from schools and places where women typically meet potential sexual partners	OCs/injectables adjHR: 1.2 (0.4–4.0)	No, unclear measurement of exposure (did not distinguish between HC methods)
Crook 2014 [21], South Africa, Uganda, Tanzania, Zambia	Cohort; RCT to assess safety and efficacy of Pro2000 candidate microbicide for HIV prevention. Enrollment 2005–2008	9385 HIV-negative women aged 16+ (18+ in South Africa and Zambia) from a range of settings. In Uganda (9% of analytic population), women were recruited as serodiscordant couples	Time-updated covariate model including time-varying exposure: DMPA adjHR 1.45 (1.09–1.93); NET-EN adjHR: 1.20 (0.84–1.69); OCs (likely COCs) adjHR: 0.90 (0.63–1.26); IPW model; DMPA adjHR: 1.49 (1.06–2.08); NET-EN adjHR: 1.31 (0.86–1.99); OC (likely COCs) adjHR: 1.00 (0.62–1.61); Additional models in Table 4 of [21]	Yes
Feldblum 2014 [23], Mozambique (Chókwè)	Cohort; to measure HIV incidence prospectively, and to assess the site's ability to enroll and retain the cohort. Data collection 2010-2012	Enrolled 479 HIV-seronegative women aged 18–35, who were sexually active in the last month, willing to adhere to study visit requirements, and planning to reside in Chókwè for duration of study, recruited from community venues with young women who engage in risky sexual behavior	OCs/injectables Crude HR: 0.4 (0.1–1.3)	No, unclear measurement of exposure (did not distinguish between HC methods; no time-varying HC exposure) and no adjustment for condom use
Wall 2015 [27], Zambia (Lusaka)	Cohort; prospective study of serodiscordant couples. Data collection 1994–2012	In this analysis, 1393 M+ F− serodiscordant couples recruited from couples voluntary counseling and testing services	HIV infections genetically linked to cohabitating male partner; Implants adjHR: 0.96 (0.29–3.14); DMPA adjHR: 1.34 (0.85–2.12); COCs adjHR: 1.39 (0.90–2.15); Linked and unlinked infections; Implants adjHR: 1.08 (0.53–2.20); DMPA adjHR: 1.19 (0.81–1.73); COCs adjHR: 1.29 (0.92–1.80)	Yes
McKinnon 2015 [25], Kenya (Nairobi)	Cohort; to estimate HIV incidence & risk factors in a program catering to FSW. Enrollment 2008–2011	Enrolled 3951 HIV-uninfected FSWs from broths, bars, clubs, and the street, as well as providing cards to clinic attendees to distribute to their peers	DMPA adjHR: 5.12 (1.98–13.22)	No, unclear measurement of exposure (no time-varying HC exposure; reference group contains unclear number of women using other HC)
Balkus 2016 [29], Malawi, South Africa, USA, Zambia, Zimbabwe	Cohort; RCT to assess safety and efficacy of BufferGel (ReProtect Inc, Baltimore, Maryland, USA) and Pro2000 (Indevus Pharmaceuticals, Lexington, Massachusetts, USA) versus placebo or no gel. Enrollment 2005–2008	Enrolled 3099 HIV-uninfected, nonpregnant women aged 18 and older who were sexually active	Injectables adjHR: 1.17 (0.70–1.96); OCs adjHR: 0.76 (0.37–1.55)	Yes
Morrison 2015 [26,38], IPD meta-analysis (and subanalysis of seven databases), Kenya, Tanzania, Uganda, South Africa	IPD meta-analysis of prospective studies	Full IPD meta-analysis included data on 37 124 sexually active women across 18 datasets; We focus on information from a subanalysis of seven studies previously unpublished studies, to avoid double-counting of component studies that are already included in our review	Full IPD meta-analysis [26]: COC adjHR: 1.07 (0.91–1.25); DMPA adjHR: 1.52 (1.27–1.82); NET-EN adjHR: 1.27 (0.99–1.61); Subanalysis of seven previously unpublished studies (two-stage random effects model) [38]; COC adjHR: 0.79 (0.38–1.64); DMPA adjHR: 1.69 (1.02–2.78); NET-EN adjHR: 1.58 (0.66–3.79)	Yes
Byrne 2016 [28] South Africa	Cohort; FRESH study – to understand mucosal immune factors associated with HIV acquisition risk. 2012–2015	Included data on 432 HIV-uninfected women aged 18–23 recruited by referral from community organizations or from community outreach	Injectables adjHR: 2.93 (1.09–7.86)	No, unclear measurement of exposure (failure to include time-varying exposure information; some women included in the injectable group did not consistently use injectables, some in comparison group used DMPA during follow-up but were considered nonusers). No adjustment for condom use, either at baseline or over time, although authors conducted a Fisher's exact test (*P* = 0.1539) to assess for differences at baseline in condom use between comparison groups
Indirect evidence from head-to-head studies				
Noguchi 2015 [30], South Africa	Cohort; to investigate the safety and efficacy of three formulations of tenofovir for HIV prevention (VOICE trial). Enrollment and follow-up 2009–2012	5029 non-HIV-infected, sexually active, nonpregnant, nonbreastfeeding women without curable genitourinary infections or abnormal renal, hematological, or hepatic functions willing to use effective contraception enrolled in RCT (952 excluded from non-South Africa sites, 936 excluded for not meeting inclusion criteria)	DMPA versus NET-EN adjHR = 1.41 (1.06–1.89)	Yes
Morrison 2015 [26] IPD meta-analysis	IPD meta-analysis of prospective studies	Authors performed a subanalysis assessing direct comparisons between HC methods among studies with pertinent data; number of included women not provided	DMPA versus NET-EN adjHR: 1.32 (1.08–1.61) (based on IPD meta-analysis of data from the following studies: [17,21,34,35,37,47,51,55,56]); DMPA versus COC adjHR: 1.43 (1.23–1.67) (based on IPD meta-analysis of data from the following studies: [17,21,24,32–37,39,42,48,51,53,55,56,60]); NET-EN versus COC adjHR: 1.30 (0.99–1.71) (based on IPD meta-analysis of data from the following studies: [17,21,34,35,37,51,55,56])	Yes

*Note:* Please refer to 2014 systematic review for detail on previously included studies [1]. adjHR, adjusted hazard ratio; adjIRR, adjusted incidence rate ratio; CI, confidence intervals; COCs, combined oral contraceptive pills; DMPA, depot medroxyprogesteone acetate; FSW, female sex worker; HC, hormonal contraception; HIV, human immunodeficiency virus; HR, hazard ratio; IPD, individual participant data; IPW, inverse probability weighted; NET-EN, norethisterone enanthate; OCs, oral contraceptive pills; POPs, progestin-only pills; RCT Randomized controlled trial.

**Table 2:** Comparison of newly included studies (published since 15 January 2014) considered ‘informative but with important limitations’, for systematic review update on use of various hormonal contraceptive methods among women at risk of HIV acquisition.

Study, study population	Number seroconverted/number analyzed, number of seroconverters by exposure group, overall HIV incidence	Interval between visits, length of f/u, loss to f/u, and whether f/u was differential by HC status	Referent group (including overall proportion of condom use in population)	Handling of condom use	Results	Summary of strengths	Summary of weaknesses
Direct evidence from HC versus no-HC studies							
Crook 2014 [21], S. Africa, Uganda, Tanzania, Zambia	382/8663 seroconverted. 265 seroconversions among women using a method of HC at baseline (146 DMPA, 69 NET-EN, 50 OCs), 117 seroconversions in women using no HC at baseline. 4.7/100 person-years	4 weekly-intersurvey interval. F/u continued for 52 weeks. Loss to f/u: 9% for DMPA, 10% for NET-EN, 11% for OCs, 10% for no HC; not differential by group	Percentage of non-HC group using each method at baseline: male or female condoms for family planning (50%), natural or traditional methods (4%), sterilization (1%), IUD (1%), no contraception (44%)	Controlled for condom use at last sex act (baseline and f/u every 4 weeks)	Time-updated covariate model including time-varying exposure: DMPA adjHR 1.45 (1.09–1.93); NET-EN adjHR: 1.20 (0.84–1.69); OC adjHR: 0.90 (0.63–1.26); IPW models; DMPA adjHR: 1.49 (1.06–2.08); NET-EN adjHR: 1.31 (0.86–1.99); OC adjHR: 1.00 (0.62–1.61); Additional models in Table 4 of manuscript	Large number of incident infections. Multisite study. Low loss to f/u. Thorough exploration of statistical methodology and sensitivity analyses, all suggesting similar results. Disaggregation of DMPA and NET-EN. Short intersurvey intervals (4 weeks). 9% of sample were serodiscordant couples. Findings consistent across sites	No information on partner's HIV status for most participants. Unable to separate POPs and COCs. Potential for residual/unmeasured confounding
Wall 2015 [27], Zambia	252/1393 seroconverted; 99 seroconversions (linked and unlinked) in women using HC (49 OC, 41 injectable, 9 implant), 153 in women using no HC. 74 seroconversions (linked) in women using HC (35 OC, 33 injectable, 6 implant), 133 in women using no HC. 8.9/100 person-years	3 month intersurvey interval (with a subset of participants followed monthly for HIV testing). Study took place over 17 years, median f/u 440 days (interquartile range 756). Loss to f/u unclear	Non-HC group comprised of individuals using condoms; copper IUD; hysterectomy, tubal ligation, or vasectomy; or no method. Proportions not described	Controlled for unprotected sex in last 3 months in analysis of linked infections. No control for condoms in analysis of linked and unlinked infections	Implants adjHR: 0.96 (0.29–3.14); DMPA adjHR: 1.34 (0.85–2.12); OCs adjHR: 1.39 (0.90–2.15); (*Note:* we display results from the analysis on incidence HIV infections genetically linked to the cohabitating male partner. Although this model had less statistical power, it was the most fully adjusted model, including controls for condom use as well as partner viral load.)	Analysis of serodiscordant couples and control for partner HIV characteristics. Large number of incident seroconversions. Included clinical characteristics of partners, such as viral load. Short intersurvey intervals (3 months). Examined presence of sperm on a vaginal swab wet prep. Long-term f/u. Conducted multiple sensitivity analyses to assess whether findings were robust to various assumptions	Various study quality components were poorly described, including: loss to f/u, number of couples with only one f/u visit, differences between exposure groups, how variables collected inconsistently over study duration were handled analytically; composition of the reference group; how information on contraceptive exposure was collected, and statistical power. Unable to separate POPs and COCs. Potential for residual/unmeasured confounding
Balkus 2016 [29], Malawi, S. Africa, Zambia, Zimbabwe (US site excluded)	106/2830 seroconverted; 88 seroconversions in women using HC (72 injectable, 15 OCs). 19 seroconversion in women using no HC. 4.07/100 person-years	Pregnancy tests monthly, HIV and contraceptive info quarterly, HSV info at baseline and study exit; 12 month f/u. Loss to f/u unclear	% of non-HC group using each method at baseline: condoms (58%), sterilization (14%), no contraceptive method (28%)	Controlled for unprotected sex at last vaginal intercourse	Injectables adjHR: 1.17 (0.70–1.96); OCs adjHR: 0.76 (0.37–1.55)	Large sample size, multisite study, short intersurvey intervals (between monthly and 3 monthly)	Didn’t differentiate between injectables or OC type. Loss to f/u unclear. No control for study arm. Potential for residual/unmeasured confounding
Morrison 2015 IPD meta-analysis [26,38] (and subanalysis of seven databases); East and Southern Africa	1830 incident seroconversions in data from full IPD meta-analysis; DMPA: 5.1/100 woman-years; NET-EN: 4.8/100 woman-years; COCs 3.4/100 woman-years; No HC 3.9/100 woman-years	Ranged from monthly to every 6 months in full IPD meta-analysis	No HC (condoms, sterilization, nonhormonal IUD, diaphragm, no modern method)	Controlled for condom use, (parameterization unspecified)	Full IPD meta-analysis [26]: COC adjHR: 1.07 (0.91–1.25); DMPA adjHR: 1.52 (1.27–1.82); NET-EN adjHR: 1.27 (0.99–1.61); Subanalysis of seven previously unpublished studies (two-stage random effects model) [38]; COC adjHR: 0.79 (0.38–1.64); DMPA adjHR: 1.69 (1.02–2.78); NET-EN adjHR: 1.58 (0.66–3.79)	IPD meta-analysis included both published and previously unpublished data. Represents the largest analysis to date of this subject, and offered a consistent approach to coding and multivariable analysis across datasets. Multisite (by nature of inclusion of studies from various settings). Extremely high statistical power permitted ability to conduct several key subgroup analyses. Numerous sensitivity analyses which generally supported overall findings (except study quality and region)	For most studies, no information on partner HIV status; variable length of intersurvey interval. Quality ranking of studies (for higher versus lower risk of bias) is necessarily subjective (and discrepant with our study quality criteria). Potential for unmeasured/residual confounding
Indirect evidence from head-to-head studies							
Noguchi 2015 [30], South Africa	207 seroconversions in 2733.7 person-years, for an incidence of 7.57/ 100 woman-years. 152/1763 person-years of DMPA (incidence: 8.62/ 100 woman-years) and 55/970.8 woman-years of NET-EN (incidence: 5.67/ 100 woman-years)	Monthly	NET-EN users	Condom use at last sex, assessed monthly	DMPA versus NET-EN adjHR = 1.41 (1.06–1.89)	Large prospective study, careful documentation of exposure to injectables, use of ACASI, adjustment for variety of time-varying covariates, monthly intersurvey intervals, head-to-head comparison may be less likely confounded by behavioral differences, multiple sensitivity analyses generally supported overall findings. Low loss to f/u	No information on partners’ HIV status. Loss to f/u differential by comparison arm. Head-to-head comparisons cannot assess whether DMPA increases risk of HIV acquisition relevant to no hormonal contraception; underlying risk of comparison group is uncertain. Potential for residual/unmeasured confounding
Morrison 2015 [26] IPD	1830 incident seroconversions in data from full IPD meta-analysis; DMPA: 5.1/100 woman-years; NET-EN: 4.8/100 woman-years; COCs 3.4/100 woman-years; No HC 3.9/100 woman-years	Ranged from monthly to every 6 months in full IPD meta-analysis	Head-to-head comparisons included: DMPA versus COC; DMPA versus NET-EN; NET-EN versus COC	Controlled for condom use, (parameterization unspecified)	DMPA versus COC adjHR: 1.43 (1.23–1.67) (17 studies included); DMPA versus NET-EN adjHR: 1.32 (1.08–1.61) (8 studies included); NET-EN versus COC adjHR: 1.30 (0.99–1.71) (8 studies included)	IPD meta-analysis included both published and previously unpublished data. Represents the largest analysis to date of this subject, and offered a consistent approach to coding and multivariable analysis across datasets. Multisite (by nature of inclusion of studies from various settings). Extremely high statistical power permitted ability to conduct several key subgroup analyses. Numerous sensitivity analyses which generally supported overall findings (except study quality and region). Head-to-head comparisons may be less likely confounded by behavioral differences	For most studies, no information on partner HIV status; variable length of intersurvey interval. Quality ranking of studies (for higher versus lower risk of bias) is necessarily subjective (and discrepant with our study quality criteria). Head-to-head comparisons cannot assess whether various HC methods increase risk of HIV acquisition relevant to no hormonal contraception; underlying risk of various comparison groups is uncertain. Potential for unmeasured/residual confounding

*Note:* Please refer to 2014 systematic review for detail on previously included studies [1]. adjHR, adjusted hazard ratio; COCs, combined oral contraceptive pills; DMPA, depot medroxyprogesteone acetate; f/u, follow up; f/u, follow-up; HC, hormonal contraception; HIV, human immunodeficiency virus; IPD, individual participant data; IPW, inverse probability weighted; IUD, intrauterine device; NET-EN, norethisterone enanthate; OCs, oral contraceptive pills; POPs, progestin-only pills.

**Table 3:** Inclusion and quality rating of publications and databases across systematic reviews and meta-analyses assessing the association of injectables (versus nonuse of hormonal contraception) with risk of HIV acquisition in women.

		Current systematic review and meta-analysis	Ralph *et al.* [19] meta-analysis	Brind *et al.* [20] meta-analysis (longitudinal data)	Morrison *et al.* [26] IPD meta-analysis
	References	Inclusion, quality rating	Inclusion	Inclusion	Inclusion/exclusion rationale, quality rating and rationale
Published manuscripts with risk estimates for injectables and HIV acquisition	Bulterys *et al.* (1994) [40]	○	○	●	Did not meet inclusion criteria; follow-up visits >6 months apart
	Ungchusak *et al.* (1996) [62]	○	○	●	Did not meet inclusion criteria; not sub-Saharan Africa
	Kilmarx *et al.* (1998) [46]	○	○	○	Did not meet inclusion criteria; not sub-Saharan Africa
	Kapiga *et al.* (1998) [44]	○	○	○	Did not meet inclusion criteria; follow-up visits >6 months apart
	Kiddugavu *et al.* (2003) [45]	○	●	●	Did not meet inclusion criteria; follow-up visits >6 months apart
	Baeten *et al.* (2007) [39] (update of [77], [54])	●	●	●	○ A
	Myer *et al.* (2007) [56]	● (6 mo estimates)	●	●	○ A, B, C
	Kleinschmidt *et al.* (2007) [47]	●	●	●	○ A, B
	Kumwenda *et al.* (2008) [48]	○	○	●	○ B
	Watson-Jones *et al.* (2009) [60]	○	○	●	○ B
	Morrison *et al.* (2010) [52] (reanalysis of [53])	●	●	●	●
	Feldblum *et al.* (2010) [41]	○	○	●	Did not meet inclusion criteria; no longitudinal data on contraception
	Reid *et al.* (2010) [58]	●	●	●	Did not meet inclusion criteria; >5% missing data for exposure
	Heffron *et al.* (2012) [42,43]	●	●	●	●
	Morrison *et al.* (2012) [55]	●	●	●	●
	Wand and Ramjee (2012) [31]	Replaced by [21]	● Duplicates [21]	● Duplicates [21]	Replaced by [21]
	McCoy *et al.* (2013) [51]	●	●	●	●
	Lutalo *et al.* (2013) [50]	○	○	δ	Did not meet inclusion criteria; follow-up visits >6 months apart; published after meta-analysis dataset closed
	Kapiga *et al.* (2013) [24]^a^	○	δ	δ	●
	Crook *et al.* (2014) [21]^a^	●	●	●	○ B
	Wall *et al.* (2015) [27]^a^	●	†	†	†
	McKinnon *et al.* (2015) [25]^a^	○	†	†	†
	Byrne *et al.* (2016) [28]^a^	○	†	†	†
	Balkus *et al.* (2016) [29]^a^	●	†	†	†
					
Data in Morrison *et al.* [26] with no associated publication specifically assessing HC-HIV	Kaul *et al.* (2004) [32]^a^	●	†	†	○ A, B
	Vallely *et al.* (2007) [33]^a^	●	†	†	○ A
	Delany-Moretlwe and Rees (2010) [34]^a^	●	†	†	○ A, B
	McGrath *et al.* (2014) [35]^a^	●	†	†	○ C, D
	Vandepitte *et al.* (2011) [36]^a^	●	†	†	○ A
	Abdool Karim *et al.* (2010) [37]^a^	●	†	†	○ D
	Van Damme *et al.* (2012) [17]^a^	●	†	†	○ D

● Included in analysis and ranked as: ‘informative but with important limitations’ (in current systematic review) or ‘lower risk of bias’ (in Morrison *et al.* [26]); indicates ‘Included’ in Ralph *et al.* [19] or Brind *et al.* [20]. ○ Included in analysis and ranked as ‘unlikely to inform the primary question’ (in current systematic review) or ‘higher risk of bias’ (in Morrison *et al.* [26]); indicates ‘not included’ in Ralph *et al.* [19] or Brind *et al.* [20]. δ Reason for noninclusion not specified. † Likely published after meta-analysis dataset closed (in Morrison 2015) or subsequent to search strategy (Brind *et al.* [20] and Ralph *et al.* [19]). A: <80% retention rate. B: Did not measure one or more of the following variables: pregnancy status, coital frequency, marital status/living with partner, or transactional sex. C: Contraceptive method measurement occurred less frequently than every 3 months. D: <10% in no–hormonal contraception comparison group.

^a^New information identified for this systematic review.
