# Supplementary material for: An updated systematic review of epidemiological evidence on hormonal contraceptive methods and HIV acquisition in women
Source: AIDS. 2016 Oct 25;30(17):2665–83. doi: 10.1097/QAD.0000000000001228 (PMC5106090; doi:10.1097/QAD.0000000000001228)

**SUPPLEMENTARY WEB APPENDIX**

**Appendix A.**

Currently, the WHO recommends no restriction (MEC Category 1) on use of the following HC methods for women at high risk of HIV: combined HCs (including combined oral contraceptive pills [COCs], combined contraceptive patches, combined contraceptive vaginal rings, combined injectable contraceptives), progestogen-only pills (POPs), and levonorgestrel (LNG) or etonogestrel (ETG) implants. Progestogen-only injectables (POIs) including DMPA and NET-EN are also classified as MEC category 1, but given the unresolved questions related to potential risk of HIV acquisition, the following clarification applies for POIs:

*“Women at high risk of HIV who are using POIs should be informed that available studies on the association between POI contraception and HIV acquisition have important methodological limitations hindering interpretation. Some studies suggest that women using POI contraception may be at increased risk of HIV acquisition; other studies have not found this association. The public health impact of any such association would depend upon the local context, including rates of injectable contraceptive use, maternal mortality and HIV prevalence. This must be considered when adapting guidelines to local contexts. WHO expert groups continue to actively monitor any emerging evidence. At the meeting in 2014, as at the 2012 technical consultation, it was agreed that the epidemiological data did not warrant a change to the MEC. Given the importance of this issue, women at high risk of HIV infection should be informed that POIs may or may not increase their risk of HIV acquisition. Women and couples at high risk of HIV acquisition considering POIs should also be informed about and have access to HIV preventive measures, including male and female condoms.”*

WHO classifies levonorgestrel (and copper) intrauterine devices (IUDs) as MEC category 2 for women at high risk of HIV, indicating that the advantages of using this method generally outweigh the theoretical or proven risks and reflecting concern about the potential increased risk of pelvic inflammatory disease with IUD use among women at high risk of STIs.

**Appendix B. Search strategy**

Our search strategy included papers published in any language, and used the following date limits: January 15, 2014 (the date on which the search strategy for our previous systematic review ended) through January 15, 2016. We also handsearched reference lists of relevant publications.

The following search strategy was performed in PubMed:

((((((hormonal AND contracepti*) OR (“hormonal methods”)) OR ((progestin* OR progestins[MeSH] OR Progesterone[MeSH] OR progestogen* OR progestagen*) AND contracept*) OR (oral contracept*) OR ((((depo OR depot) AND medroxyprogesterone) OR depomedroxyprogesterone OR depo OR depot OR dmpa OR “Sayana Press” OR “net en” OR “NET-EN” OR “norethisterone enanthate” OR norethisterone-enanthate OR Medroxyprogesterone 17-Acetate[MeSH]) AND (contracept* OR inject*)) OR “Depo Provera” OR “Depo-Provera” OR (((levonorgestrel OR etonogestrel) AND implant) OR (uniplant OR jadelle OR implanon OR nexplanon OR norplant OR norplant2 OR sino-implant)) OR (contraceptives, postcoital[MeSH] OR (contracept* AND (emergency OR postcoital OR “post coital”)) OR “ulipristal acetate” OR “Plan B” OR mifepristone) OR ((levonorgestrel AND (intrauterine devices[MeSH] OR iud OR iucd OR ius OR “intrauterine system” OR “intra-uterine system” OR “intrauterine device” OR “intra-uterine device”)) OR mirena) OR ((combin* AND inject* AND contracept*) OR ((“once a month” OR monthly) AND inject* AND contracept*) OR (cyclofem OR lunelle OR mesigyna OR “cyclo provera” OR cycloprovera)) OR ((((contraceptive devices[MeSH] OR contraceptive agents[MeSH]) AND ring) OR nuvaring OR “nuva ring”)) OR ((((contraceptive devices[MeSH]OR contraceptive agents[MeSH]) AND patch) OR “ortho evra” OR orthoevra)) AND ("HIV Seropositivity"[ MeSH] OR "HIV"[MeSH] OR "HIV Infections"[MeSH] OR “HIV” OR "Acquired Immunodeficiency Syndrome"[MeSH] OR “HIV transmission”)) OR (injectable contracepti* HIV) OR (oral contracepti* HIV))) AND ("2014/01/15"[EDAT] : "2016/01/15"[EDAT]))

The following search strategy was performed in Embase:

hormonal AND contracepti* OR 'hormonal methods' OR (progestin* OR 'progestins' OR 'progesterone' OR progestogen* OR progestagen* AND contracept*) OR ('oral' AND contracept*) OR ((depo OR depot) AND 'medroxyprogesterone') OR depomedroxyprogesterone OR depo OR depot OR dmpa OR 'sayana press' OR 'net en' OR 'net-en' OR 'norethisterone enanthate' OR (('medroxyprogesterone' AND '17-acetate') AND (contracept* OR inject*)) OR (('levonorgestrel' OR 'etonogestrel') AND 'implant') OR 'uniplant' OR 'jadelle' OR 'implanon' OR 'nexplanon' OR 'norplant' OR norplant2 OR 'sino implant' OR (contraceptives, AND postcoital) OR (contracept* AND ('emergency' OR postcoital OR 'post coital')) OR 'ulipristal acetate' OR 'plan b' OR 'mifepristone' OR ('levonorgestrel' AND 'intrauterine' AND 'devices') OR 'iud' OR 'iucd' OR ius OR 'intrauterine system' OR 'intra-uterine system' OR 'intrauterine device' OR 'intra-uterine device' OR 'mirena' OR (combin* AND inject* AND contracept*) OR (('once a month' OR monthly) AND inject* AND contracept*) OR 'cyclofem' OR 'lunelle' OR 'mesigyna' OR 'cyclo provera' OR 'cycloprovera' OR (('contraceptive' AND 'devices') OR ('contraceptive' AND agents) AND ring) OR 'nuvaring' OR 'nuva ring' OR (('contraceptive' AND 'devices') OR ('contraceptive' AND agents) AND patch) OR 'ortho evra' OR orthoevra AND ('hiv seropositivity' OR 'hiv' OR 'hiv infections' OR 'acquired immunodeficiency syndrome' OR 'hiv transmission') OR (injectable AND contracepti* AND 'hiv') OR ('oral' AND contracepti* AND 'hiv') AND [humans]/lim AND [15-1-2014]/sd NOT [15-1-2016]/sd AND ([article]/lim OR [article in press]/lim OR [erratum]/lim OR [letter]/lim OR [review]/lim)

**Appendix C. Quality assessment criteria**

Studies were considered “unlikely to inform the primary question” if they had one or more of the following flaws:

- No adjustment for any measure of condom use unless authors report trivial differences comparing estimates from models including and not including condom use, or
- Unclear measurement of exposure to HC, including one or more of the following:
  - Failure to include time-varying analysis of HC exposure, if appropriate (e.g., time-varying analysis may not be necessary for studies with extremely short follow-up periods).
  - Failure to provide separate estimates for different types of HC methods (e.g., OCs or injectables or implants). We did not exclude studies that grouped together different formulations of a particular method (e.g., combined depot-medroxyprogesterone acetate (DMPA) and norethisterone enanthate (NET-EN) into a single exposure category).
  - Comparison group included a substantial or unclear number of users of another HC method (except in an intentional head-to-head comparison of a specific HC method versus another specific HC method).
  - The interval of time between study visits (“intersurvey interval”) was longer than 6 months, with contraceptive use measured only at each interval endpoint (and thus providing only limited information about possible contraceptive switching during the intersurvey interval). (Note: if variation in length of intersurvey interval occurred within an individual study, such that some intervals were 6 months or less and other intervals were longer than 6 months, we included only data from intervals that were 6 months or less).

Studies considered “informative but with important limitations” had none of the flaws described above.

Note: Elements new to this framework since the 2014 version are underlined.

**Appendix D. Supplementary Figures.**

**Appendix D, Figure 1. Use of oral contraceptives (versus non hormonal contraception) and HIV acquisition (all 24* studies, regardless of quality)**

**
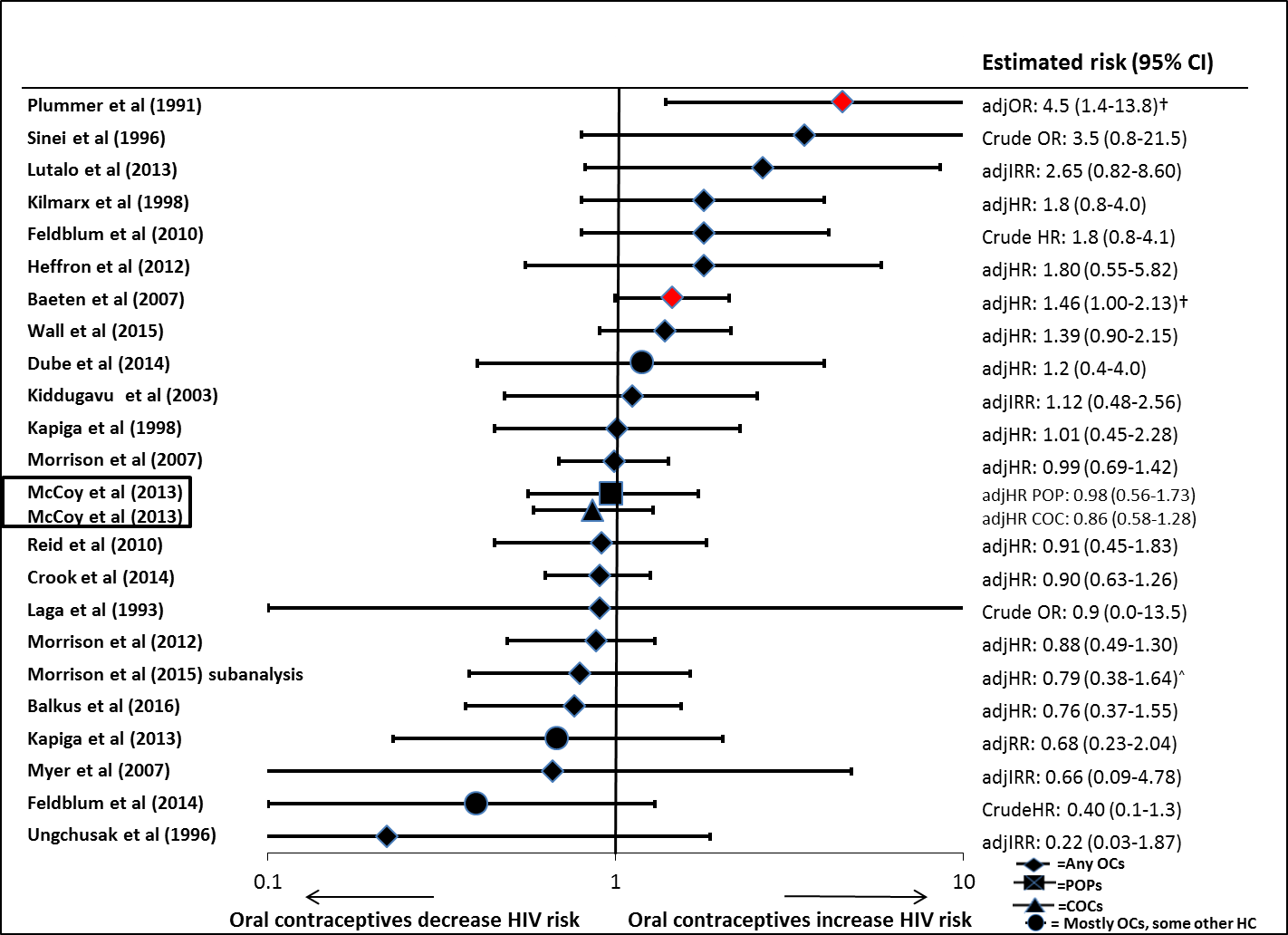
**

Error bars show 95% CIs. Studies are arranged in order of decreasing magnitude of risk estimate, except if a single study disaggregated POPs and combined oral contraceptives [COCs], in which case both estimates are adjacent (as indicated by a box around the study identifiers). Graph does not display estimates from marginal structural models (MSM). Crude OR, crude odds ratio. adjOR, adjusted odds ratio. adjIRR, adjusted incidence risk ratio. adjHR, adjusted hazard ratio. * Data from Saracco et al not shown, risk could not be calculated since no seroconversions occurred in the hormonal contraception group. †Analysis showed statistically significant findings at p=.05 (marker also displayed in red). ^ Unpublished estimates from a sub-analysis of Morrison 2015 meta-analysis, restricted to pooled analysis using databases not previously used to publish estimates on hormonal contraceptive methods and HIV acquisition risk.

**Appendix D, Figure 2. Use of injectable contraceptives (versus non hormonal contraception) and HIV acquisition (all 24 studies, regardless of quality)**

**
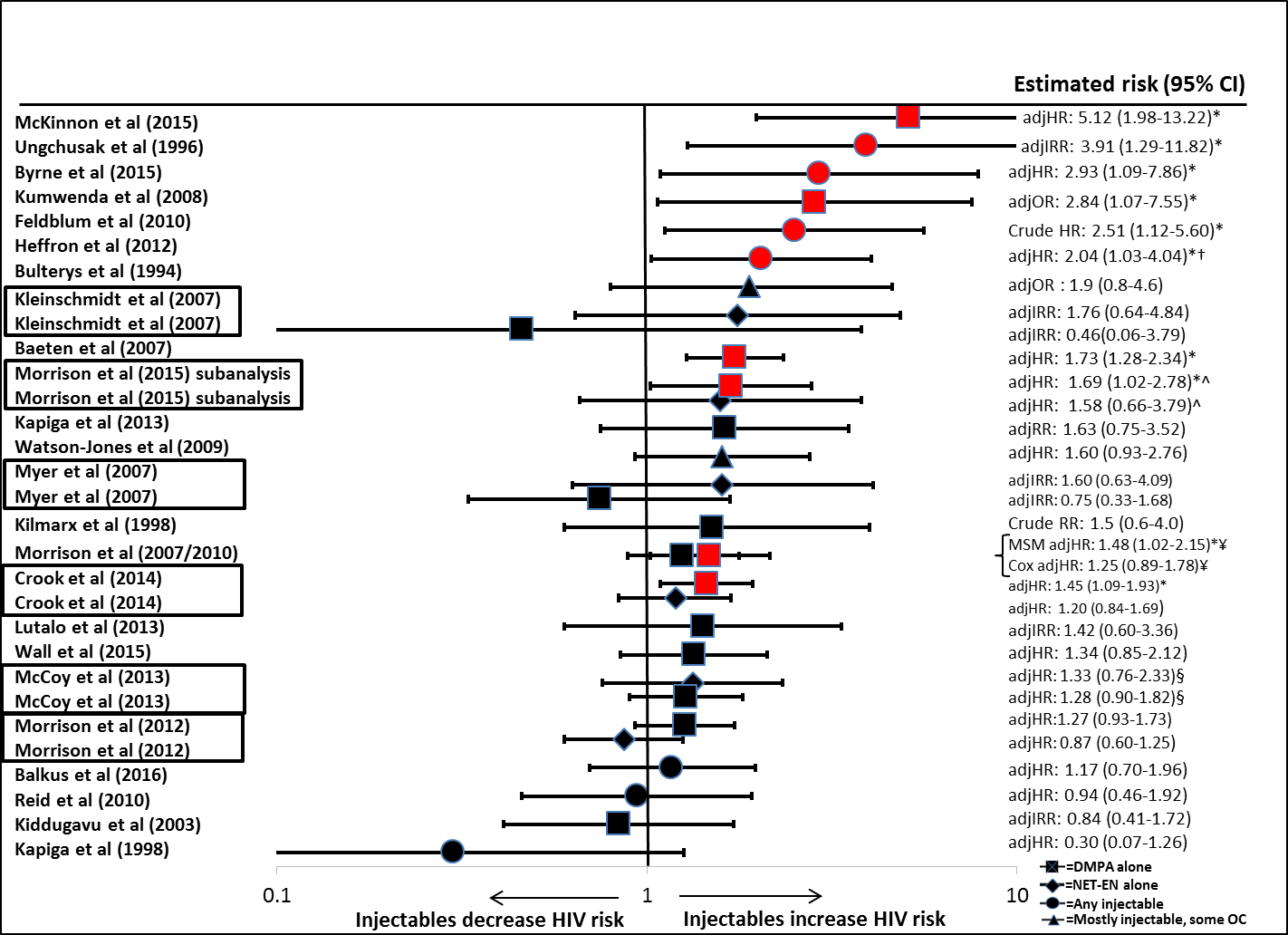
**

Error bars show 95% CIs. Studies arranged in order of decreasing magnitude of risk estimate. Graph does not display estimates from marginal structural models (MSM), except where use of such models resulted in different conclusion regarding statistical significance; in such cases, estimates from both models are displayed on a single line (also identified by bracket signs). adjIRR, adjusted incidence risk ratio. adjHR, adjusted hazard ratio. *Analysis showed significant findings at p=.05 (marker also displayed in red). †Estimate for Cox model taken from slightly updated analysis which controlled for total number of unprotected sex acts. ^ Unpublished estimates from a sub-analysis of Morrison 2015 meta-analysis, restricted to pooled analysis using databases not previously used to publish estimates on hormonal contraceptive methods and HIV acquisition risk. ¥ Different statistical models adjusted for slightly different confounders. §Unpublished estimates disaggregated by injectable type.

**Appendix D, Figure 3. Use of implants (versus non hormonal contraception) and HIV acquisition (all 3 studies, regardless of quality)**

**
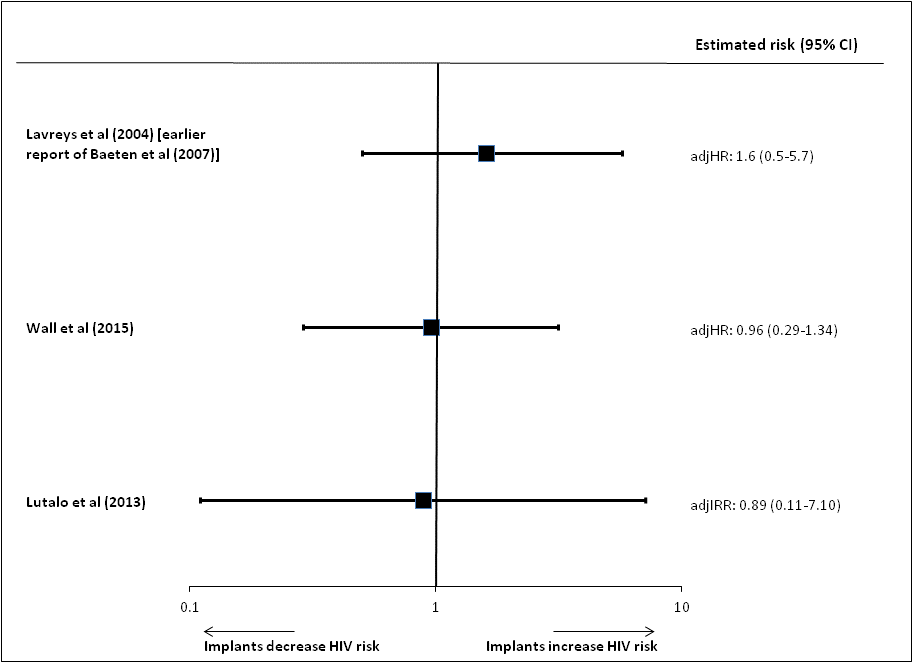
**

Error bars show 95% CIs. Studies arranged in order of decreasing magnitude of risk estimate. adjIRR, adjusted incidence risk ratio. adjHR, adjusted hazard ratio.

**Appendix D, Figure 4. Use of implants (versus non hormonal contraception) and HIV acquisition, among 2 studies considered informative but with important limitations**

**
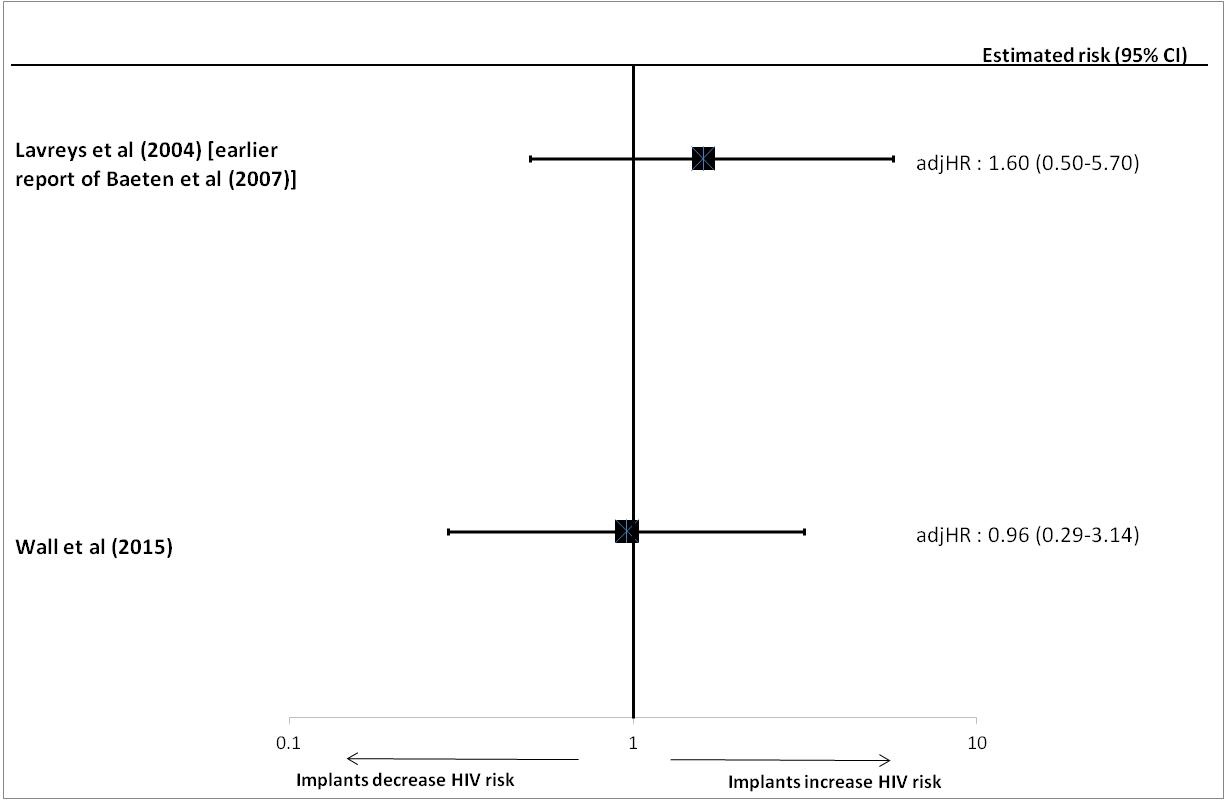
**

Error bars show 95% CIs. Studies arranged in order of decreasing magnitude of risk estimate. adjHR, adjusted hazard ratio.

**Appendix D, Figure 5: DMPA/HIV meta-analyses**


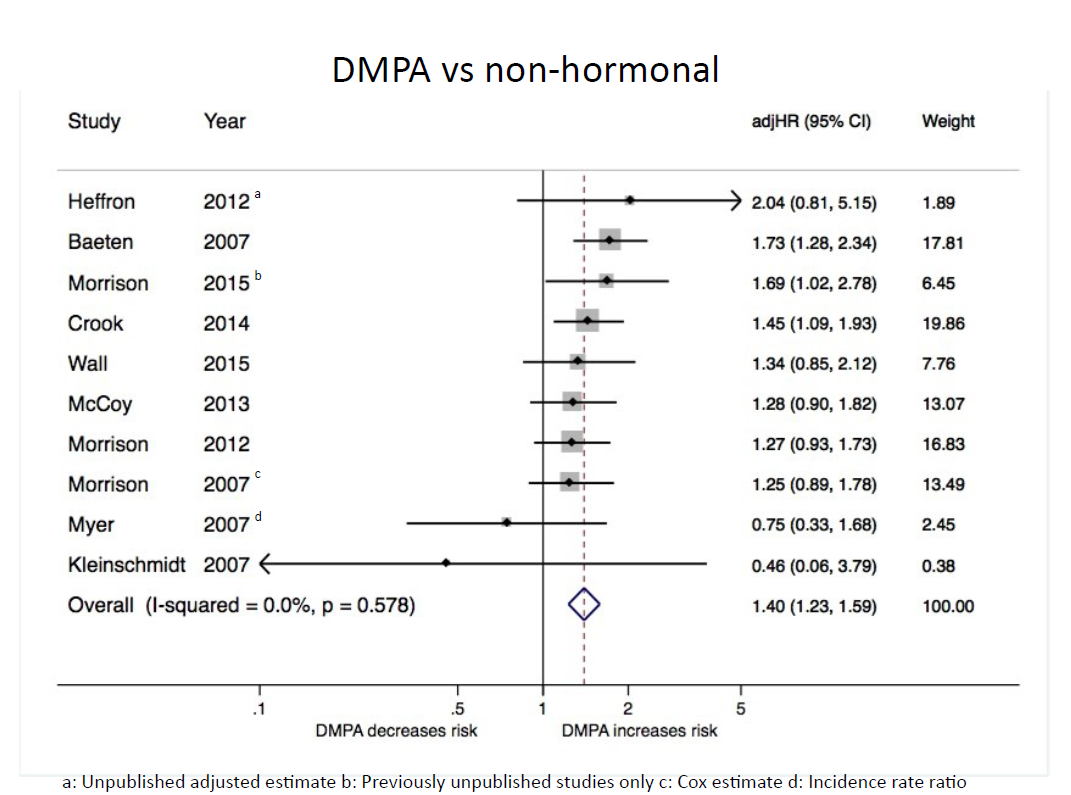

Supplement: Supplemental Digital Content [file aids-30-2665-s001.doc]
